# Goblet cell carcinoid of the appendix – diagnostic challenges and treatment updates: a case report and review of the literature

**DOI:** 10.1186/s13256-018-1789-6

**Published:** 2018-09-24

**Authors:** Gregory Gilmore, Kristin Jensen, Shreyas Saligram, Thomas P. Sachdev, Subramanyeswara R. Arekapudi

**Affiliations:** 1 Department of Medicine, Veterans Affairs Central California Health Care System, University of California San Francisco, 2615 E Clinton Ave, Fresno, CA 93703 USA; 2 Department of Pathology, Veterans Affairs Palo Alto Health Care System, Stanford Hospital and Clinics, Palo Alto, CA 94304 USA

**Keywords:** Goblet cell carcinoid, Appendix, Chemotherapy

## Abstract

**Background:**

Goblet cell carcinoid is a rare but distinct entity of appendiceal tumors which is a hybrid or mixed tumor consisting of both epithelial (glandular) and neuroendocrine elements containing goblet cells. This entity is important to recognize and appropriately grade as it tends to be more aggressive than typical carcinoid tumors, often presenting with metastatic disease. As a result, the 5-year overall survival is 14–22% in stage III–IV disease. GCC therefore warrants more aggressive surgical and medical (chemotherapy) interventions than typical carcinoid tumors. Through this case report we give a brief update on GCC pathological features, staging, surgical management, and review the literature as a guide to indications for chemotherapy and choice of agents.

**Case presentation:**

We present the case of a 77-year-old Caucasian man with a history of stage I adenocarcinoma of transverse colon status post transverse colectomy who was incidentally found on surveillance colonoscopy to have an abnormal appendiceal orifice lesion. A biopsy revealed an appendiceal goblet cell carcinoid and he underwent a right hemicolectomy which revealed a pathologic stage III GCC for which he received eight cycles of adjuvant chemotherapy with capecitabine.

**Conclusions:**

It is essential that patients who have tumors > 2 cm, are pT3 or pT4, have higher grade histology with signet ring (Tang grade B or grade C), locally advanced, or with positive surgical margins on appendectomy undergo a right hemicolectomy. Although there is no category 1 evidence, consensus recommendations are that patients with stage II (particularly Tang B and C) and stage III GCC be offered adjuvant chemotherapy with a regimen based on 5-fluorouracil, as these patients are known to have high rates of relapse.

## Background

Primary cancers of the appendix are quite rare representing less than 1% of all gastrointestinal malignancies with an annual incidence of approximately 1.2 cases per 100,000 people in the USA [1]. Although a small organ, tumors of the appendix can develop into cancers with significant morphologic diversity and thus are further classified into adenocarcinoma, carcinoid (neuroendocrine tumors; NETs), mucinous tumors, signet ring cell tumors, and goblet cell carcinoids (GCCs). GCC is exceedingly rare accounting for approximately 14–19% of primary appendix cancers [1, 2]. It is a distinct entity as a hybrid or mixed tumor consisting of both epithelial (glandular) and neuroendocrine elements containing goblet cells. GCC is more common in Caucasians with a mean age at diagnosis of 58, and there is no known difference in incidence between males and females [1, 3–5]. At this time there are no known or established risk factors that increase a person’s probability of developing GCC [6]. As with most tumors of the appendix, GCC frequently presents with acute abdominal pain and clinical findings of appendicitis in 50–60% of cases [7–9]. It is often diagnosed incidentally during appendectomy or ileocecal resection and confirmed on surgical pathology [10]. Prognosis is very good if diagnosed at stage I or II, but significantly worsens in stage III or IV disease (5-year overall survival 22% and 14% respectively) [11]. Disease-specific 5-year survival for all patients presenting with GCC is 58–81% [2, 12, 13]. Due to the rarity of GCC there are no randomized trials or clinical guidelines for treatment but adjuvant 5-fluorouracil (5-FU)-based regimen is recommended for stage III or stage IV disease [8, 9, 13].

This rare entity is important to recognize and appropriately diagnose as the biology of this disease is more aggressive than typical carcinoid tumor and treatment needs to be tailored accordingly. Unfortunately, there are limited data to guide adjuvant treatment and most appropriate chemotherapy regimen. In this case report we review the presently available literature to help guide treatment decisions for those patients diagnosed as having GCC.

## Case presentation

We present a case of a 77-year-old Caucasian man with past medical history of stage I adenocarcinoma of transverse colon status post laparoscopically assisted segmented transverse colectomy in April 2014. Other medical history included type 2 diabetes, hypertension, hypothyroidism, and benign prostatic hypertrophy. Medications at the time of diagnosis included aspirin, metformin, lisinopril, and levothyroxine. His family history included lung cancer in his father who was a tobacco smoker. Our patient was a former tobacco smoker but denied history of alcohol or drug abuse and had no history of occupational or chemical exposure. He presented for follow-up screening colonoscopy approximately 2 years later in July 2016 at which time he was asymptomatic. His Eastern Cooperative Oncology Group (ECOG) performance status was grade 1. On clinical examination he was afebrile, mildly hypertensive with blood pressure 146/81, heart rate 78, respiratory rate of 16 with oxygen saturation of 96% on room air. He had normal cardiac rate and rhythm, and no abnormal breath sounds on respiratory examination. His abdomen had normal bowel sounds on auscultation, was soft and non-tender without distension. A neurologic examination demonstrated normal neurologic function without sensory deficits and normal muscle strength.

**Fig. 1:**
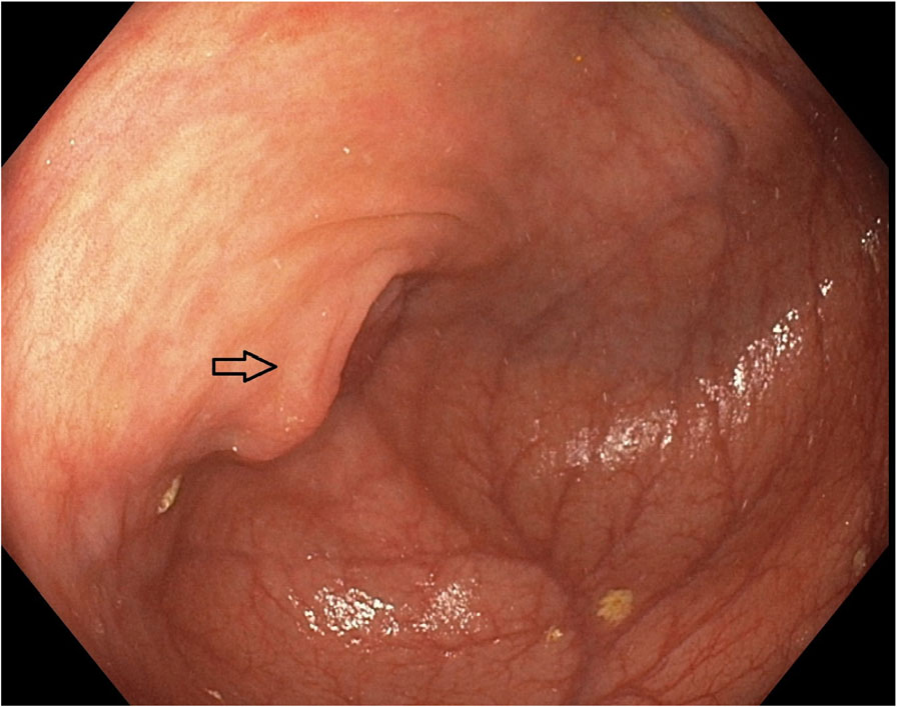
Image from colonoscopy showing the abnormal-appearing appendiceal orifice (indicated by *arrow*) from which biopsies were taken

On colonoscopy, he was found to have an abnormal-appearing appendiceal orifice which was biopsied; pathology was suggestive of mucinous adenocarcinoma with signet ring cell features versus a goblet cell-type carcinoid tumor of the appendix (Fig. 1). The appendiceal orifice appeared normal on previous colonoscopies in March and December of 2014. Pre-colonoscopy complete blood count (CBC) revealed white blood cell (WBC) count of 5.7 10^3^/uL (reference range 4–11), hemoglobin 13.9 g/dl (reference range 14–17) with mean corpuscular volume (MCV) of 82.3 fL (reference range 80–94), and platelet count of 171 K/mm^3^ (reference range 150–400). Pre-colonoscopy basic chemistry including sodium, potassium, chloride, bicarbonate, and creatinine were all within normal limits.

**Fig. 2:**
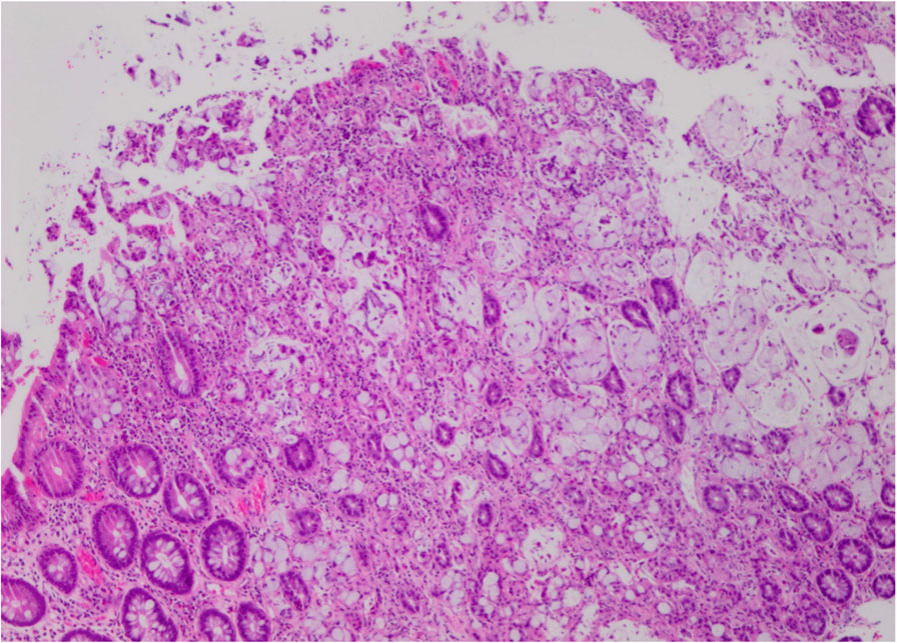
At low magnification, the tumor is seen infiltrating normal colonic glands, as nests and small rounded clusters of cells, many of which are distended by mucin. (Hematoxylin and eosin)

**Fig. 3:**
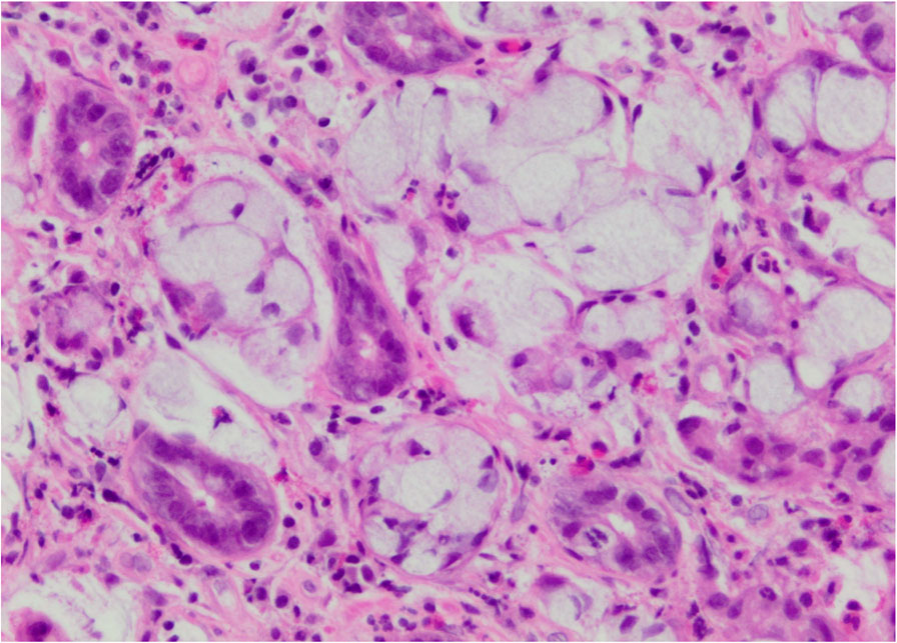
Higher magnification of the tumor highlights the mucin as well as the cytologically bland nuclei compressed to the edges of the cells. (Hematoxylin and eosin)

**Fig. 4:**
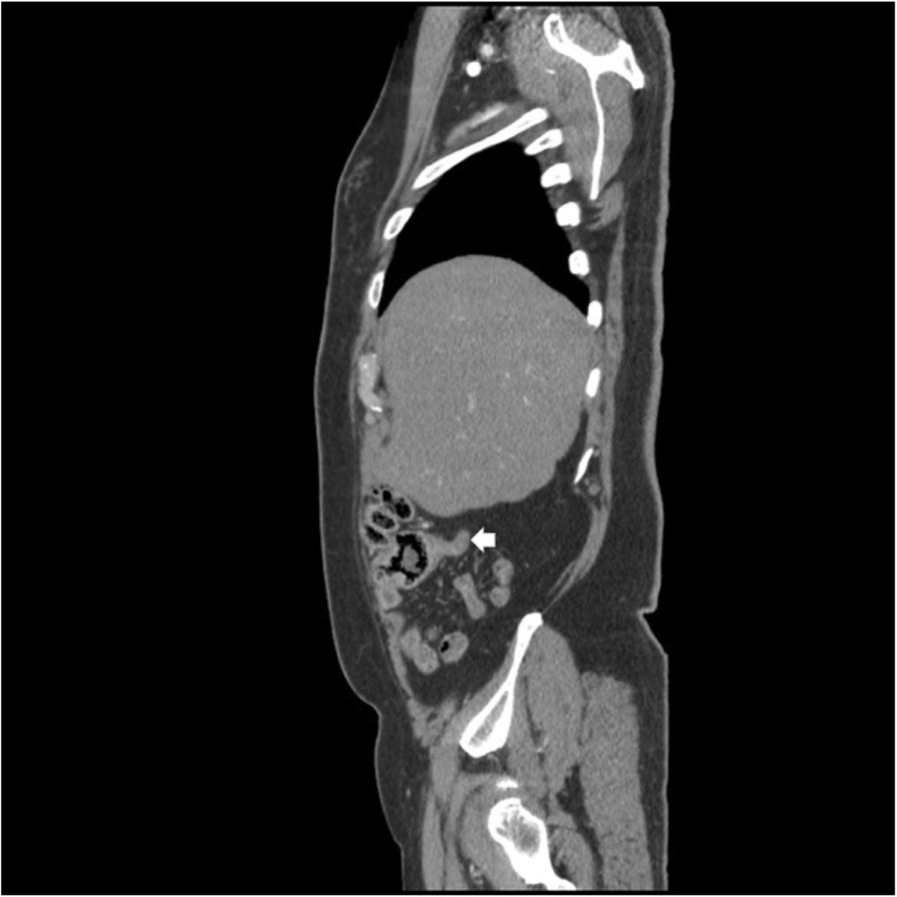
Computed tomography scan of the chest, abdomen, and pelvis showing a thickened appendix at 12 mm in diameter as indicated by *arrow*

On histological examination the tumor was present as infiltrative small nests and clusters of cells with small nuclei compressed by abundant cytoplasmic mucin vacuoles, giving a signet ring appearance (Figs. 2 and 3). Given the location of the lesion at the appendiceal orifice, the diagnosis of goblet cell carcinoid was strongly suspected, but definitive diagnosis was deferred to complete resection. Further laboratory workup with tumor markers and neuroendocrine markers revealed carcinoembryonic antigen (CEA) of 3.1 ng/ml (reference range 0.0–3.1) and chromogranin A, and 24-hour urine 5-hydroxyindoleacetic acid (5-HIAA) within normal limits. A computed tomography (CT) scan of his chest, abdomen, and pelvis showed a thickened appendix (12 mm) without evidence of fat stranding (Fig. 4). There was no significant lymphadenopathy, no colonic masses seen, and no evidence of distant metastatic disease.

**Fig. 5:**
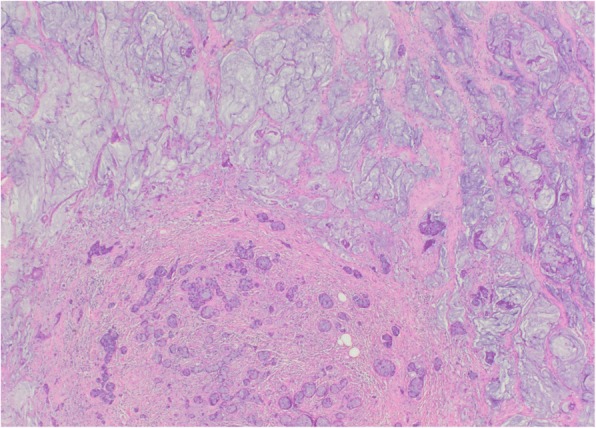
The *lower half* of the field indicates what was once the appendiceal lumen, but which now shows small nests of cells distended by mucin. In the *upper half and right side* of the field, the nests have coalesced to form large infiltrating pools of mucin, some of which contain cells “floating” within the mucin. (Hematoxylin and eosin)

**Fig. 6:**
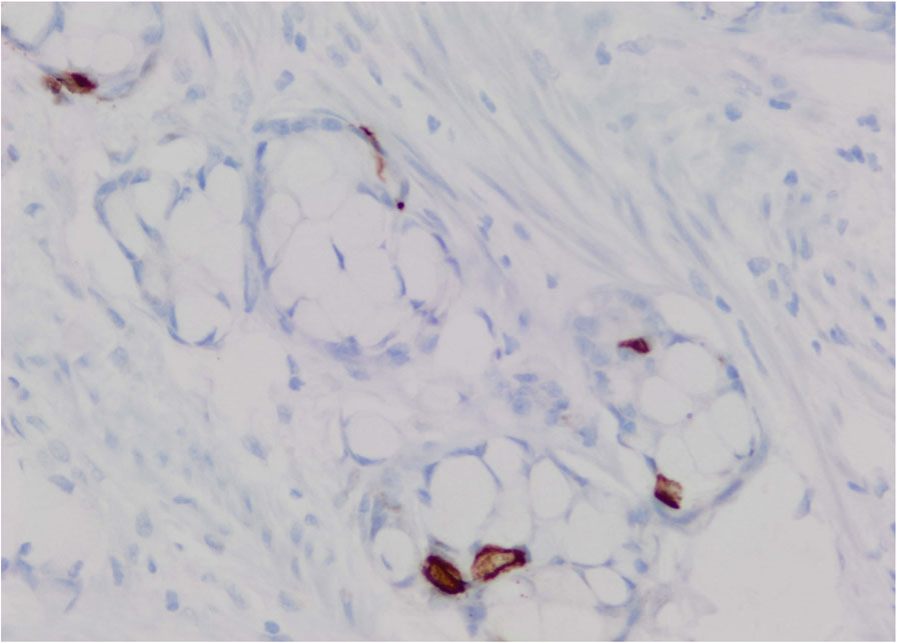
An immunohistochemical stain for synaptophysin highlights scattered peripheral endocrine cells within the tumor nests. (Synaptophysin)

After surgical evaluation, he underwent a right hemicolectomy in August 2016. Both specimens from colonoscopy and right hemicolectomy were sent for expert consultation. On pathologic review, the bulk of the tumor involved the appendix, essentially obliterating the lumen, with diffuse spread into the mesoappendix and serosal adipose tissue. Both perineural and lymphovascular invasion were noted. Six of 14 lymph nodes harbored metastatic carcinoma. In areas of the appendiceal wall, the nests of signet ring cells coalesced into pools of mucin containing “floating” cells, indicating frank mucinous carcinoma, so-called adenocarcinoma ex-goblet cell carcinoid (Fig. 5), Tang group B. Immunohistochemistry for synaptophysin highlighted scattered occasional peripheral endocrine cells, as is characteristic of goblet cell carcinoid (Fig. 6). The final pathologic staging of the patient's tumor was pT3 N1 M0, stage III as per American joint committee on cancer staging manual, 7th edition [14].

Postoperatively, we discussed treatment options including adjuvant chemotherapy; our patient was initially against adjuvant chemotherapy due to prior experiences with family members, but he agreed to it after the rationale was explained. He was given adjuvant capecitabine with a goal of eight cycles. Given his age, the first four cycles of capecitabine were given at a 25% dose reduction of 1500 mg twice daily for days 1–14 every 21 days. As he tolerated therapy well, the dose was increased to 2000 mg twice daily for days 1–14 every 21 days for cycles five to eight. He completed eight cycles of capecitabine and tolerated treatment well other than mild hand and foot syndrome which developed during the last two cycles. A follow-up CT scan at 6 and 12 months after completion of adjuvant chemotherapy showed no evidence of recurrent disease. A repeat colonoscopy at 1 year from original diagnosis was also negative for any malignant-appearing lesions. We are continuing surveillance with history and physical with CEA every 3 months, and CT of his chest, abdomen, and pelvis every 6 months for the first 2 years and then annually for up to 5 years. At the current time he remains disease free at 2 years from time of diagnosis.

## Discussion

We presented the case of a 77-year-old Caucasian man who had a history of early stage I colon cancer and was later diagnosed as having a second primary stage III GCC, Tang group B. He underwent a right hemicolectomy followed by adjuvant chemotherapy with the orally administered fluoropyrimidine capecitabine for eight cycles. Most patients with Tang group B or Tang group C GCC have metastatic disease at time of diagnosis [13]. Fortunately, this patient had stage III GCC at diagnosis due to the fact it was found incidentally on surveillance colonoscopy. Due to limited literature in regard to adjuvant treatment options for a Tang group B or C patient without metastatic disease, it was challenging to develop an evidence-based treatment plan.

Goblet cell carcinoid is typically diagnosed postoperatively after an appendectomy or ileocecal resection and is confirmed by a pathologist in the post-surgical specimen. It is important to correctly identify tumors as GCC and categorize appropriately by Tang’s classification as particularly higher grade GCCs exhibit more aggressive behavior and this should influence postoperative management decisions [13, 15]. The overall prognosis for GCC falls between that of appendiceal adenocarcinoma, which has a poorer prognosis, and NETs, which has a better prognosis [1, 3].

The differential diagnosis for GCC includes adenocarcinoma of appendix, signet ring cell tumors, carcinoid tumors, and mucinous-benign tumors of the appendix. GCC is an unusual entity, histologically distinct from typical carcinoid. Conventional carcinoids are typically cellular, and are composed of relatively small uniform cells arranged in moderately sized solid nests, cords, or ribbons. While mucinous differentiation may be seen in conventional carcinoids, it is typically a focal finding. Immunohistochemical staining for synaptophysin or chromogranin in a conventional carcinoid will highlight sheets of cells, nearly every cell in the tumor, in contrast with the rare scattered peripheral endocrine cells seen in goblet cell carcinoid. Once a goblet cell carcinoid has become frankly malignant, most authors advocate using the term “carcinoma (or adenocarcinoma) ex-goblet cell carcinoid” to avoid any potential confusion with conventional carcinoid tumor. The plethora of proposed and historic names for goblet cell carcinoid (mucinous carcinoid, adenocarcinoid, and crypt cell carcinoma) has contributed to the confusion surrounding this entity.

There are several different classification/staging systems used for GCC including the 2010 World Health Organization (WHO) classification for appendix tumors, the 2010 AJCC (TNM classifications) staging, and recently proposed Tang *et al.* classification specific for GCC of the appendix [8]. The AJCC stages tumors as stage I (T1, N0, M0), stage II (T2/T3, N0, M0), stage III (any T, N1, M0), and stage IV (any T, any N, M1) [14]. The Tang classification uses histologic features of the tumor at the primary site to classify GCC tumors into three groups. The following groups are designated using the histologic features which include the arrangements of goblet cells, degree of atypia, and degree of desmoplasia: Typical GCC (group A), adenocarcinoma ex-GCC, signet ring cell (group B), and adenocarcinoma ex-GCC, poorly differentiated (group C). As demonstrated by Tang *et al.*, tumors classified moving from group A to C represent progressively more aggressive phenotypes and worse prognosis with all patients in group C presenting with metastatic disease [13].

Our patient was staged by AJCC (TNM) staging as T3, N1, M0, stage IIIB. The tumor obliterated the lumen of his appendix with nests/clusters of signet ring cells coalesced into pools of mucin, indicating frank mucinous carcinoma. It was thus classified by Tang classification as group B, adenocarcinoma ex-GCC, signet ring cell.

Due to the rarity of GCC, there are no *Category 1*-based guidelines from large randomized control trials on which to base treatment decisions. The mainstay of treatment for non-metastatic disease is surgical resection. However, the extent of surgical resection with appendectomy versus right hemicolectomy is debated. Since many GCCs are found incidentally after appendectomy, the need for further complete oncologic resection (that is, right hemicolectomy) is an important question.

Both the North American and European Neuroendocrine Tumor Societies recommend right hemicolectomy as standard first-line treatment for GCC even after appendectomy due to high risk of metastases and improvement in prognosis [4, 8, 16]. However, in several published analyses, there is evidence to suggest limited or no benefit of right hemicolectomy, primarily in patients with low grade and/or limited disease burden. A meta-analysis of 13 studies including 100 total patients demonstrated a failure rate of 7% with appendectomy alone versus 10% in extended resection (*p* = 0.29); the authors concluded no benefit of right hemicolectomy in patients with localized disease with low grade histology [17]. Several other small studies evaluating extent of surgical resection in GCCs have suggested there is no benefit to right hemicolectomy in those with small (< 1 cm), localized, low grade tumors without high risk features such as positive resection margins [18, 19].

In a retrospective analysis of Surveillance, Epidemiology, and End Results (SEER) data evaluating 3137 patients with appendiceal NETs (typical NETs, typical GCC, and signet ring cell adenocarcinoma), after adjusting for age, stage, and histology, there was no significant survival benefit for right hemicolectomy versus appendectomy for typical NETs (*p* = 0.21) or typical GCC (*p* = 0.94). However, in those with signet ring cell adenocarcinoma histology, they found a statistically significant benefit in survival for right hemicolectomy versus appendectomy alone (*p* = 0.01) [20]. In addition, analysis by Tang *et al.* demonstrated that histology rather than size of tumor should be used as a determining factor to decide the extent of oncologic resection (appendectomy versus right hemicolectomy) with higher grades (groups B and C) benefiting from more extensive resection. Therefore, based on these data, it is reasonable to consider appendectomy alone in those patients with tumor < 2 cm and localized to appendix with negative surgical margins, those with typical GCC group A histology, and those with pT1 or pT2 tumors. For all other patients which include those with tumors > 2 cm, locally advanced stage, positive margins, histology with signet ring group B or group C, or pT3 or pT4 tumors it is recommended to perform a right hemicolectomy [8, 11, 13, 21].

Owing to the rarity of GCC, we do not have randomized control trials or evidenced-based guidelines for choice of systemic chemotherapy. The available data are primarily anecdotal for clinician experience or published small case series. Since metastatic GCC most resembles that of colon adenocarcinoma, the selection of adjuvant chemotherapy has been extrapolated from colorectal adenocarcinoma with recommendations for regimens based on 5-FU. The most commonly used regimens historically are FOLFOX (5-FU, leucovorin, oxaliplatin) or FOLFIRI (5-FU, folic acid, irinotecan). In general, chemotherapy is recommended for select stage II and all stage III and IV disease, as well as in the setting of recurrence [8].

A retrospective review of the Mayo Clinic database from 1984 to 2004 had a prospective follow-up of 57 patients with GCC. Of these, 27 patients received chemotherapy with primarily regimens based on 5-FU which showed a trend toward mean survival benefit but it was not statistically significant. The mean survival for combined stage II to III patients who received chemotherapy was 47 months with chemotherapy versus 32 months with no chemotherapy (*p* = 0.383). For stage IV patients, mean survival was 39 and 29 months, respectively (*p* = 0.281) [11]. In another UK, single center study of patients with confirmed GCC, 18 patients received chemotherapy, 16 with curative intent. The most commonly received systemic chemotherapy regimens were either FOLFOX or single agent capecitabine. The results of this study showed no improvement in disease-free survival (DFS) (*p* = 0.870), and, in fact, patients who received adjuvant chemotherapy had a shorter DFS (21.3 versus 75.9 months). This finding is probably due to selection bias with patients with more advanced disease receiving adjuvant chemotherapy [7]. Most recently, in an article based on their institutional experience and review of available literature, *Clift et al.* proposed that chemotherapy be offered to all patients with stage II GCC or a primary tumor classified as Tang B/C, and any stage III/IV patients treated with curative intent [22]. Additional case series and retrospective reviews reporting different choices of chemotherapy regimens and their outcomes are detailed in Table 1.

**Table 1:** Goblet cell carcinoid outcomes by different chemotherapy regimens

Study	Type of study	*N*	Stage	Histology	Treatment	CR	PFS	Overall survival
Garin *et al*. [23] 2002	Case report	1	IV	Adenocarcinoid	5-FU + LV (LV5FU2) × 1 cycle followed by FOLFOX 4 × 9 months	Yes (at 8 months)	3 years	–
Pham *et al.* [11] 2006	Retrospective review	57	I – 8 II – 20 III – 6 IV – 23	Goblet cell carcinoid	5-FU + LV-based chemotherapy (*N* = 27, Stage II–IV)	–	–	Cx versus No Cx Stage II–III Mean (months): 47 versus 32 months (*p* = 0.383) 5 year (%): 43 versus 56 Stage IV Mean (months): 39 versus 29 (*p* = 0.281) 5 year (%): 26 versus 0
Toumpanakis *et al.* [24] 2007	Retrospective review	15	I–IV	Goblet cell appendiceal carcinoid	RH in all patients without metastasis. Stage IV (3) 2- VP-16 + cisplatin 1–5-FU + cisplatin +streptozotocin.	–	11/15 no disease (median follow-up 30 months)	Median 30 months follow up: − 11 alive with no disease – 1 alive with metastatic disease – 3 died of metastatic disease (9–14 months)
Tang *et al.* [13] 2008	Retrospective review	63	I – 2 II – 18 III – 3 IV – 40 Tang group A – 30 B – 26 C – 7	Goblet cell carcinoid	FOLFOX FOLFIRI (33/63) Stage III (2/3) Group B (14/26)	Group A – 86% Group B – 15% Group C – 0%	DSS % (Tang groups) 3 years Group A – 100% Group B – 85% Group C – 17% 5 years Group A – 100% Group B – 36% Group C – 0%	–
Bilen *et al.* [25] 2013	Case series	1	Metastatic	Poorly differentiated adenocarcinoma arising from goblet cell carcinoid	FOLFOX followed by CRS and HIPEC (mitomycin-C)	pCR	Disease free at 7 months	–
Clift *et al*. [22] 2018	Retrospective review	21	I – 1 II – 10 III – 5 IV – 5 Tang group A – 8 B – 10 C – 3	Goblet cell carcinoid	Appendectomy: 12 (completion RH: 8) RH: 6 Other resections: 3 6 received adjuvant cx with CAPOX – 5 dead of disease, 1 alive with no disease	–	DFS: 1-year: 94.7% 3-year: 74.2% 5-year: 74.2%	Mean OS (1-, 3-, and 5-year OS) 80.3 months (79.4%, 60%, and 60%) Group A: 73.1 months (85.7%, 85.7%, 51.4%) Group B: 83.7 months (all 66.7%) Group C: 28.5 months (66.7%, 66.7%, not reached)

*5-FU* 5-fluorouracil, *CAPOX* capecitabine/oxaliplatin, *CR* complete response, *CRS* cytoreductive surgery, *Cx* chemotherapy, *DFS* disease-free survival, *DSS* disease specific survival, *FOLFIRI* 5-FU/LV/irinotecan, *FOLFOX* 5-Fu/LV/oxaliplatin, *HIPEC* hyperthermic intraperitoneal chemotherapy, *LV* leucovorin, *OS* overall survival, *pCR* pathologic complete response, *PFS* progression-free survival, *RH* right hemicolectomy, *VP-16* etoposide

Based on a review of the literature, our patient was offered adjuvant chemotherapy given he was stage IIIB and classified as Tang group B. The choice of capecitabine single agent versus a multi-agent regimen was decided based on his age and, primarily, his preference for oral treatment and minimal toxicities.

The overall survival for patients with GCC varies based on different series and classifications or staging systems used. Overall prognosis is good for patients with early stage disease but much poorer for those who present with late stage disease. Based on Tang’s classification for groups A, B, and C, the mean overall survival was 199 months, 43 months, and 31 months, respectively. The 5-year overall survival statistics were 100%, 36%, and 0% respectively. It was also noted that a large percentage (63%) of patients with GCC presented with stage IV disease. The 5-year overall data based on AJCC (TNM) staging system is 100% for stage I, 76% for stage II, 22% for stage III, and 14% for stage IV [13].

## Conclusions

GCC is a rare but distinct entity of appendiceal tumors. It is essential to accurately diagnose GCC as it is more aggressive in nature than typical carcinoid tumor, and often presents with metastatic disease. Right hemicolectomy is recommended for tumors > 2 cm, pT3 or T4, higher grade histology with signet rings, or with positive surgical margins on appendectomy. Lastly, despite lack of category 1 evidence, consensus recommendations are patients with stage II (particularly Tang B and C) and stage III GCC should be offered adjuvant chemotherapy with a regimen based on 5-FU.
